# Covert contraceptive use among women attending a reproductive health clinic in a municipality in Ghana

**DOI:** 10.1186/s12905-016-0310-x

**Published:** 2016-06-06

**Authors:** F. Baiden, G. P. Mensah, N. O. Akoto, T. Delvaux, P. C. Appiah

**Affiliations:** Epidemiology Unit, Ensign College of Public Health, Kpong, ER Ghana; Faculty of Public Health and Allied Sciences, Catholic University College of Ghana, Fiapre, BAR Ghana; Unit of HIV/AIDS Policy, Institute of Tropical Medicine, Antwerpen, Belgium; Municipal Health Directorate, Ghana Health Service, Sunyani, BAR Ghana; Public Health Department, Institute of Tropical Medicine, Antwerp, Belgium

**Keywords:** Covert, Secret, Contraceptive, Method, Family planning, Ghana Africa

## Abstract

**Background:**

Covert contraceptive use (CCU) in sub-Saharan Africa is an indication of women’s inability to exercise autonomy in their reproductive choices. The aim of this study was to assess the prevalence and determinants of CCU among a sample of FP clients in a municipality of Ghana.

**Methods:**

We conducted a mixed method study among women attending a public reproductive health clinic in Sunyani, a city of over 250,000 inhabitants in Ghana. An initial survey inquired into sociodemographic characteristics, use of family planning (FP) methods and partner awareness of contraceptive use. The predictors of CCU were explored using logistic regressions. We used the findings to develop a guide which we applied in-depth interviews and focus group discussions with attendants at the same facility. Qualitative data analysis was conducted using a framework approach.

**Results:**

We interviewed 300 women, 48 % of whom were aged between 26–33 years. The injectable was the most widely used method (56 %). The prevalence of CCU was 34 %. In multivariate analysis, single women were more likely to practice CCU than married or co-habiting women (Adjusted OR = 12.12, 95 % C.I. 4.73–31.1). Muslim and traditionalist women were similarly more likely to practice CCU than non-Muslim, non-traditionalist (Adjusted OR = 4.56, 2.29–9.06). Women who preferred to have their first or next child in 4 or more years from the time of the interview were more likely to be in CCU than women who intended to have children within 4 years of the interview (2.57; 1.37–4.83). Single women saw in covert use a statement of their social autonomy. To succeed in CCU, women wished that clinic attendance cards would not be given to them to keep at home. Though many participants saw in CCU a source of anxiety, they expected health workers to consider it and uphold confidentiality in the provision of services.

**Conclusions:**

Covert contraceptive use was high in this municipality and being single was the strongest predictor of the practice. Providers of FP services should reflect on how to adequately address the challenges faced by women who practice CCU.

## Background

Covert contraceptive use (CCU) is the practice of using a family planning (FP) method without the knowledge of the partner [1, 2]. In contemporary literature, it invariably refers to the use of such methods by a woman without the knowledge of the male partner. The practice is common among women in sub-Saharan Africa (SSA) and it has been linked to the degree to which women are able to exercise autonomy in their reproductive choices [3–5]. It is also said to represent misperceptions between partners about each other’s views on the use of contraceptives and aspirations regarding family size [1]. Since the 1994 International Conference on Population and Development, the empowerment of women to enable them to exercise the optimal reproductive health choices has been an issue of major concern [6]. Covert contraceptive use is considered to be symptomatic of the lack of ability of a woman to freely exercise her reproductive rights [1, 3]. The extent to which interventions to empower women in SSA has impacted on the practice of CCU remains unknown.

The practice of CCU has been studied in different sociocultural settings in SSA. The prevalence is found to be high among women in male-dominated, rural societies. Estimates of between 6 % and over 50 % have been reported [1, 7–9]. Among the factors found to be associated with the practice are the fear of partner violence, withdrawal of economic support and partner accusations of infidelity [10–13]. A major theme that runs through the literature on CCU is the extent and quality of spousal communication on FP [14–16]. Correlations have been established between the frequency of spousal communication and contraceptive use in general, with women more inclined to adopt a covert approach where there is infrequent or no discussions about FP with the partner [1, 17, 18].

The desire of women to use contraceptive covertly is said to reflect in their choice of FP methods [19–21]. Methods such as the injectable, IUD, pills and implants are considered to lend to discreet use by women while methods such as the condom (male or female), foaming tablet and diaphragm require the awareness of the male partner for effective use [22, 23]. An effect of the quest to achieve covert contraceptives use is the possibility that women may be compelled to use FP methods that do not necessarily meet their biological needs.

Ghana was one of the first countries in SSA to adopt an explicit comprehensive population policy in 1969. Among the key provisions of the policy is the promotion of FP as an important part of the country’s development agenda. Evidence from the Ghana Demographic and Health Survey (GDHS) 2008 indicates that 98 % of all women and 99 % of all men know of at least one method of contraception. The use of modern FP methods has increased more than threefold (from 5 to 17 %) between 1988 and 2008. In the same period, total fertility rate (TFR) dropped from 6.4 to 4.0, putting Ghana among countries with the lowest TFRs in SSA [24]. In spite of these achievements the level of unmet FP need (estimated at 35 %), unwanted pregnancies and unsafe abortions remain high [25, 26]. Pervasive gender inequities and norms regarding the subordination of women give men greater power than women in the exercise of reproductive rights in Ghana [27, 28].

Very little is known about current practices regarding CCU in Ghana. This is particularly the case of single women as they have been excluded in most previous studies. Very little is known about how the experience of single women compares with that of women in presumably stable relationships. An improved understanding of the practice of CCU is needed to inform the design of interventions that will promote the ability of women to exercise their contraceptive choices with autonomy, and without strain on their relationships. In the case of single women, it is also needed to inform the design of interventions to reduce unwanted pregnancies and unsafe abortions [29, 30]. We conducted a study among women attending a FP clinic in a municipality in Ghana to establish the prevalence of CCU, and to identify and explain the factors that influence the practice.

## Methods

### Study design and site

A cross-sectional mixed-method study was conducted at the Reproductive Health Clinic (RHC) at the Sunyani Municipal Hospital, in the Brong Ahafo Region of Ghana. This RHC is part of the public health unit of the Sunyani Municipal Health Directorate and offers comprehensive reproductive health services that include counselling and provision of a wide range of FP methods. The RHC serves an urban population of about 250,000 people, 25 % of whom are women of child-bearing age. The majority of women in Sunyani are petty traders, peasant farmers and workers in the formal sector. Over 90 % of women in the municipality earn less than three hundred Cedis a month (equivalent to $78 U.S.) [31].

### Quantitative component (Survey)

All women who were using at least one modern FP method and attended the RHC between March and April 2012 were targeted to be interviewed with a questionnaire that inquired into sociodemographic characteristics, type and duration of use of the method and male partner awareness of the use of a method. The question to elicit CCU was posed as “Is your partner aware that you are using a modern FP method?” The questionnaire was administered by a trained research assistant who was a non-health worker. To assess the extent to which women were economically-empowered, a question was asked about the proportion of their total household cost that was supported by their income. Respondents were also asked about their willingness to recommend the use of modern family planning methods to their friends, and whether their own use of modern FP methods conflicted with their religious beliefs. The interviews were conducted in the respondent’s preferred language. Although they were conducted within the premises of the RHC, privacy was ensured with the use of a private room within the facility. A target sample size of 300 women was set to afford 95 % confidence level in estimating the prevalence of CCU within a margin of error of 5 %, assuming a covert use prevalence of 26 %.

Data from the completed questionnaires were double entered into computer using Epi-DATA software. It was then exported into Stata version 12 for analyses that were descriptive and exploratory of the relationship between CCU, sociodemographic characteristics, and, practices and intentions regarding FP use. Covert contraceptive use was defined as the practice where a women used a modern FP method without the knowledge of the male partner. Bivariate (unadjusted) and multivariate (adjusted) logistic regression analysis was performed to identify predictors of CCU. At both bivariate and multivariate levels, an association was considered to be significant if the two-tailed *P*-value was less than 0.05. Only variables that were statistically significant in the bivariate analysis (Table 1) were included in multivariable model (Table 2). The findings are reported in two tables that show factors related to the sociodemographic characteristics of respondents and factors that are related to FP intentions and practices. Exploratory analyses was also performed to describe in further detail the characteristics of groups found to be significantly likely to practice CCU (e.g. single women).

**Table 1:** Bivariate analysis of the determinants of covert and non-covert contraceptive use among women attending a reproductive health clinic in Sunyani, Ghana

Variable		Total	Covert use		Unadjusted	*P*-value
			Yes (%)	No (%)	O.R. (95 % CI)^a^	
Age	≤25 years	74 (25 %)	34 (32 %)	40 (21 %)	1.86 (1.08–3.19)	0.02
	>25 years	226 (75 %)	71 (68 %)	155 (79 %)	1.00	
Marital status	Single	76 (26 %)	49 (47 %)	27 (14 %)	5.25 (2.88–9.56)	<0.01
	Married or co-habitation	218 (74 %)	56 (53 %)	162 (86 %)	1.00	
Highest educational levels	Tertiary	79 (29 %)	35 (35 %)	44 (26 %)	1.55 (0.91–2.67)	0.11
	Less than tertiary	189 (71 %)	64 (65 %)	125 (74 %)		
Religion	Muslims & traditionalist	70 (36 %)	38 (36 %)	32 (17 %)	2.80 (1.59–4.93)	<0.01
	Christians	225 (64 %)	67 (64 %)	158 (83 %)	1.00	
Place of residence	Within metropolis	227 (76 %)	77 (73 %)	150 (77 %)	0.83 (0.47–1.43)	0.49
	Outside metropolis	73 (24 %)	28 (27 %)	45 (23 %)	1.00	
Number of children	None	59 (20 %)	30 (29 %)	29 (15 %)	2.29 (1.27–4.12)	<0.01
	At least a child	241 (80 %)	75 (71 %)	166 (85 %)	1.00	
Contribution to household cost	>50 %	41 (14 %)	18 (17 %)	23 (12 %)	1.24 (0.61–2.53)	0.10
	25–50 %	127 (42 %)	36 (34 %)	91 (47 %)	0.63 (0.37–1.06)	
	<25 %	132 (44 %)	51 (46 %)	81 (41 %)	1.00	
Contraceptive method currently used by respondent	Implants	36 (12.8 %)	12 (11.9 %)	24 (13.3 %)	1.08 (0.50–2.34)	0.10
	Combined oral contraceptive pills	54 (19.2 %)	27 (26.7 %)	27 (15.0 %)	2.17 (1.15–4.09)	
	Intrauterine device	23 (8.2 %)	9 (8.9 %)	14 (7.8 %)	1.39 (0.57–3.44)	
	Injectable	168 (76 %)	53 (66 %)	115 (81 %)	1.00	
Respondent’s duration of use of modern FP method	>12 months	127 (42 %)	38 (36 %)	89 (46 %)	0.82 (0.45–1.49)	0.13
	7–12 months	88 (29 %)	38 (36 %)	50 (26 %)	1.47 (0.79–2.73)	
	0–6 months	85 (28 %)	29 (28 %)	56 (28 %)	1.00	
Respondents’ desired interval to next or first child	≥4 years	70 (23 %)	34 (33 %)	36 (19 %)	2.13 (1.22–3.71)	0.01
	<4 years	228 (67 %)	70 (67 %)	158 (81 %)	1.00	
Respondent’s willingness to recommend FP to friends	Yes	285 (95 %)	97 (92 %)	188 (96 %)	0.45 (0.16–1.29)	
	No	15 (5 %)	8 (8 %)	7 (4 %)	1.00	
Respondents considers FP to conflict with her religious beliefs	Yes	97 (32 %)	41 (39 %)	56 (29 %)	1.56 (0.96–2.63)	0.06
	No	203 (68 %)	64 (61 %)	139 (71 %)	1.00	
Respondent’s main source of FP information^b^	Friends	109 (36 %)	47 (45 %)	62 (32 %)	N/A	N/A
	Mass media	167 (56 %)	58 (55 %)	109 (56 %)		
	Husband	24 (8 %)	0 (0 %)	24 (12 %)		

^a^OR denotes odds ratio for contraceptive covert use and CI denotes confidence interval

^b^FP—family planning

**Table 2:** Multivariate analysis^a^ of the determinants of covert and non-covert contraceptive use among women attending a reproductive health clinic in Sunyani, Ghana

Variable		Total	Covert use		Adjusted^b^
			Yes (%)	No (%)	O.R. (95 % CI)^c^
Age	≤25 years	74 (25 %)	34 (32 %)	40 (21 %)	0.63 (0.26–1.54)
	>25 years	226 (75 %)	71 (68 %)	155 (79 %)	1.00
Marital status	Single	76 (26 %)	49 (47 %)	27 (14 %)	12.12 (4.73–31.1)
	Married or co-habitation	218 (74 %)	56 (53 %)	162 (86 %)	1.00
Religion	Muslims & traditionalist	70 (36 %)	38 (36 %)	32 (17 %)	4.56 (2.29–9.06)
	Christians	225 (64 %)	67 (64 %)	158 (83 %)	1.00
Number of children	None	59 (20 %)	30 (29 %)	29 (15 %)	1.22 (0.45–3.26)
	At least a child	241 (80 %)	75 (71 %)	166 (85 %)	1.00
Contraceptive method currently used by respondent	Implants	36 (12.8 %)	12 (11.9 %)	24 (13.3 %)	1.16 (0.47–2.88)
	Combined oral contraceptive pills	54 (19.2 %)	27 (26.7 %)	27 (15.0 %)	1.66 (0.76–3.64)
	Intrauterine device	23 (8.2 %)	9 (8.9 %)	14 (7.8 %)	0.96 (0.32–2.82)
	Injectable	168 (76 %)	53 (66 %)	115 (81 %)	1.00
Respondents’ desired interval to next or first child	≥4 years	70 (23 %)	34 (33 %)	36 (19 %)	2.57 (1.37–4.83)
	<4 years	228 (67 %)	70 (67 %)	158 (81 %)	1.00

^a^Includes only variables that were statistically significant at a *P*-value of <0.05 in the bivariate model (Table 1)

^b^ *P*-value for the multivariate logistic regression model was <0.01

^c^OR denotes odds ratio for contraceptive covert use and CI denotes confidence interval

### Qualitative component (In-depth Interviews and Focus Group Discussions)

The findings of the survey were used to develop a guide for conducting in-depth interviews (IDIs) and focus group discussions (FGDs) to further explore the practice of CCU and contextualize the findings of the survey. The guide explored the concept of autonomy among single women, the influence of religion, birth spacing and spousal communication and how they affect CCU. A research assistant with experience in qualitative data collection conducted the IDIs and FGDs. A purposive sample of 36 clients at the same facility were asked to participate in 8 IDIs and 4 FGDs between January and February 2015. The participants were selected based on the fact that they were clients to the facility. We actively sought to include women who were current users of a modern FP method. However a few (less than 10 %) of the participants in the FGDs were women who were not on any method but were at the clinic to discuss the various options with the FP counsellors. All the participants however needed to willingly volunteer to participate in the IDIs and FGDs. The number of IDIs and FGDs was dictated by resource availability and time limitation. No woman participated in both IDIs and FGDs.

Except in the case of IDIs with two women who declined, all IDIs and FGDs were tape-recorded, transcribed and typed into computer. Both women who declined tape-recording were Muslims who feared they could be heard on tape by a third party who might compromise their CCU. They were however agreeable to notes being taken and reported. We used a framework approach to analyse the data. The framework was based on the findings of the survey and the guide we used in the IDIs and FGDs. The objective was to obtain information that will help to contextualize the findings. Emergent themes were also sought and noted. The analysis, including coding were performed by two researchers who worked independently but later compared and reconciled their findings.

## Results

**Fig. 1:**
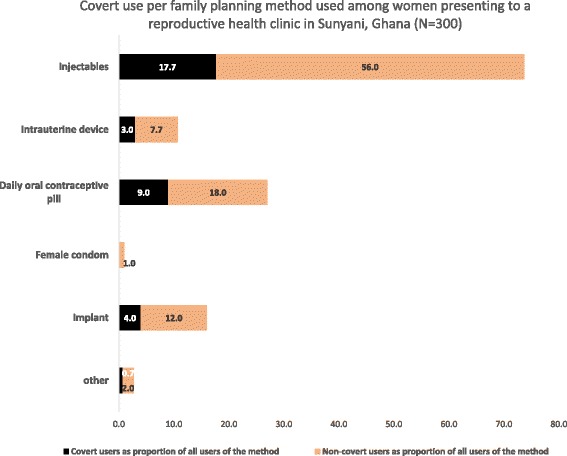
Covert use per family planning method used among women presenting to a reproductive health clinic in Sunyani, Ghana (*N* = 300)

We interviewed 300 women in the survey. This represented 99 % of the women we approached. The only woman who refused to be interviewed gave no reason for her refusal. About 97 % of the respondents were aged between 18 and 41 years, and nearly half (48 %) were between the ages of 26 and 33 years. The majority (74 %) were either married (58 %) or co-habiting (16 %). About 26 % of women interviewed were single (including divorced or separated). While a fifth (20 %) of women had no children, 42 % had two or more. Of those who were single, divorced or separated, 45 % had at least one child. Only 26 % of respondents had had education beyond high school. About 9 % of them had had no formal education. The majority (75 %) were Christian. Muslims and traditionalists (worshippers of other deities) constituted the remaining 25 %. The majority (76 %) of women lived within the Sunyani metropolis. Only 14 % of women indicated that they made financial contributions that met at least 50 % of their total household costs. The FP methods used by participants included the injectable (56 %), oral contraceptive pill (18 %) implant (12 %) and intrauterine device (8 %). Overall about a third (34 %) of women admitted to CCU. This however varied according to the type of FP method being used Fig. 1.

Being single, including being divorced or separated (*n* = 6) was the strongest independent predictor of CCU. Single women were more likely to be in CCU than married or co-habiting women (Adjusted O.R. = 12.12, 95 % C.I. 4.73–31.05). Table 2 Single women with no children were also more likely to practice CCU than married or co-habiting women with children (O.R. = 4.35, 2.16–8.75). Among single women alone, covert use was not significantly influenced by whether a woman had a child or not (O.R. = 1.50, 0.55–4.04). (data not shown)

Uncertainty about the future of relationships emerged in the FGDs and IDIs as an important underlying reason why single women practised CCU. They indicated they did not feel obliged to make that disclosure since the relationship was not marriage. A comment by a participant that CCU was statement that expressed a woman’s autonomy was met with enthusiastic affirmation by the majority of participants in the FGDs.

“*Once the man has not married me, I am my own person and I do not need to let him know*”

*“If he hasn’t married me yet, then I am myself, and what will help me is what I will do”*

Consistent with the above, there was near unanimity that such autonomy got compromised once women got married. During the IDIs, at least two women spoke about how CCU enabled single women to, as and when they found necessary, feign pregnancy and use it as a basis for claiming money from their partners. This money was claimed for the purpose of procuring an abortion. When this was brought in the FGDs, it met with the approval of most participants. No participant countered the opinion. The approval of participants took the form of enthusiastic nodding and giggling, with older women appearing eager to indicate their agreement. A corroborative comment described a situation where this could happen.

“*You may have boyfriend who is stingy and the only way you will be able to get money off him will be to feign pregnancy and appear to agree with him to abort it. How will you be able to do that if you let him know you are using a FP method*?”

In multivariate analysis, the other sociodemographic characteristic that was significantly associated with CCU was religion (Table 2). Muslim and women who identified themselves as traditionalist were significantly more likely to practice CCU than non-Muslim, non-traditionalist (Adjusted O.R. = 4.56, 2.29–9.06). This effect of religion was evident during the IDIs and FGDs as many women (Muslims and non-Muslims alike) recounted how much the use of FP was frown upon in Muslim and traditional societies in the municipality. The two women who declined to have their interviews recorded spoke passionately about possible dire consequences for them if members of their families or close associates of their partners found out that they had attended the FP facility and were actually using a method. Christian women also recounted similar possible adverse repercussions for their relationships. This was however to a lesser extent. The direction of the association between religion and CCU did not surprise any of the participants in the IDIs and the FGDs.

The age of women (Adjusted O.R. = 0.63, 0.26–1.54) and the number of children they had (Adjusted O.R. = 1.22, 0.45–3.26) did not significantly predict CCU in multivariate analysis. This was not particularly surprising to participants in the IDIs and FGDs. A few participants opined however that they expected younger women and women who had about four children or more to be more likely to practice CCU. This was linked to the findings regarding singles and disagreement with spouse over the desired target number of children respectively.

Among the factors related to FP intentions and practices that were explored, only the desired length of time to a next (or first) pregnancy was found in multivariate analysis to be significantly associated with the likelihood of CCU (Table 2). Women who preferred to have their first or next child in 4 or more years from the time of the interview were more likely to be in CCU than women who intended to have children within 4 years of the interview (Adjusted O.R. = 2.57; 1.37–4.83). Related to this, women in the IDIs and FGDs conveyed the impression that the desires of women regarding the appropriate number of years in between pregnancies often differed from that of their partners. It was suggested that men often wanted to have all their desired number of children within a few years while they (women) wished to space them well enough to be able to recover fully from the physiological effects of the preceding pregnancy. This was said to be more the case when male partners were much older.

Although the use of the COC was associated with CCU in bivariate analysis, this was not found to be an independent predictor in multivariate analysis. The practice of CCU was not found to be significantly associated with the use of any particular FP method (Table 1). Discussions around this during the IDIs and the FGDs revealed that the choice of particular FP methods was dictated more by factors that were not explored in this study. This included client and or provider convenience, advice received from the nurse who attended to them at the time of the visit, the availability of the method at the facility at the time of a visit, the availability on-site of a nurse who was trained to offer particular methods. These were typically put as follows

*“The side effect of the injection is minimal compared to the other methods”*

*“Yes, I use the injectable not because I wish to hide it from my husband, but because I want to avoid the risk of forgetting.”*

*“The nurse told me it was the best”*

*“I was told the nurse who could put the IUD was not around. So I opted for what was available”*

It was generally felt however that methods such as the injectable, implant and intrauterine device were easier to use covertly than the oral contraceptive pill.

Overall, women did not consider CCU to be an ideal practice since, among other reasons it denied them the support of their partners. Such support included reminders of dates of clinic appointments, compassion and understanding when hormonal contraceptives caused disruption to menstrual flow and interfered with sexual pleasure. It also put them at considerable risk of the loss of trust in case the partner found out. Women appeared to be compelled into CCU by the circumstances of their relationships which may it difficult for them to bring up issues about contraceptives use with their partners. Underlying this concern was a fear that they could be suspected of infidelity. The fact that they could not anticipate partner support also constrained women in their choice of methods as they had to choose methods that were least likely to disrupt the pattern of menstrual flow or sexual pleasure. From both personal and non-personal experiences, participants mentioned some of the consequences of unsuccessful CCU to be physical abuse, extramarital affairs, withdrawal of financial support and threats of divorce.

*“I am not ok with using contraceptive without the knowledge of the my partner but I do not want my husband to take another wife”*

In order to achieve covert contraceptive use, women appeared to go to great lengths. A participant recounted how she travels nearly 40 km to come to the RHC just so his husband does not find out that she is visiting a FP clinic. Another women recounts how after each visit to the clinic she passed by her mother’s house to drop her RHC attendance card before proceeding to where she lives with the husband.

On what women wanted the attitude of health workers to be towards CCU, there was unanimity about the expectation that FP nurses and counsellors need to appreciate the challenge clients faced in this regard. They felt that nurses and counsellors needed to consider it a duty to support them to achieve their intention of covert use. Some went further to suggest ways through which health workers could assist. It included the clinic making a policy to keep all patients cards at the facility and not giving any materials to clients to send home.

*“They should assist us by keeping the folders in the hospital instead of giving to us to take home”*

*“They should have a high sense of confidentiality, because some of them are staying in our areas and may come into contact with relatives of ours”*

## Discussion

This study has used a mixed-methods approach to explore the practice of CCU among women attending a reproductive health clinic in a municipality in Ghana. The CCU prevalence of 34 % found in this population compares with 7 % in urban Zambia [20], 31 % in urban Mali [32] and over 50 % in rural northern Ghana [9]. The wide variations in prevalence in different settings may be the result of the different methodological approaches used in the different studies, and the lack of a standard definition for CCU [33, 34]. While some studies obtain data interviewing both partners (either separately or simultaneously), [33, 35–37] most others do so interviewing only women [32, 38]. Similarly while some studies obtain information through community-based methods ([1, 36, 37], others do so through facility-based approaches [34, 38]. For a practice that is dictated by strong sociocultural norms, such differences in approaches are likely to lead to varying estimates of the extent of the practice. The use of such prevalence values and comparisons across different settings should to be done with due regard for the setting in which the studies were conducted and the methods that were used.

From the sentiments expressed by women in this study, it appears CCU is not a practice that women readily admit to. It is thus probable that the CCU prevalence of 34 % found in this study may well be an underestimation of the true extent of the practice. In any case, such a high level of CCU cannot be ignored by the health system and needs to be taken into account in the planning of reproductive health services in this and similar health facilities in Ghana. There is the need for more in-depth studies into the practice with the view to developing interventions that will assist women who are uncomfortable with covert use and need assistance to engage their spouses to be supported while women who wish to maintain covert use are assisted to do so. Currently reproductive health services in Ghana maintain no routine records on the extent of covert use. Women distressed by spousal disapproval of contraceptive use have no formal avenue for seeking assistance within the health service. Greater advocacy is needed to bring the issue of CCU to the fore. Interventions need to be developed to respond directly to the challenge that it poses to the right of women to have unfretted access to the full range of options in FP methods, and to be open about it if they wish to do so. The other attributes of the practice of CCU such as the influence of religion and birth spacing intentions are consistent with established knowledge about the adoption of FP methods by women in Ghana [1, 27, 32]. They lends credence to suggestions that the social constructs around contraceptive use in Ghana are rooted in sociocultural norms and male dominance remains a formidable theme [18, 27, 39].

Although CCU has rarely been mentioned in relation to single women, it has been anticipated that the problems associated with communication and reproductive decision-making could still apply [1, 40]. In spite of this, contraceptive use by single women remains one of the least studied areas in FP. The finding in this study that single women are more likely to practice CCU than married women and the insight provided in the follow-up qualitative component gives indication that there is some uniqueness in the behaviour of single women regarding their use of contraceptives. While it is conceivable that women in less binding relationships would practice CCU as a statement of their autonomy, to consider that it could be a means for achieving financial gain in a relationship was an unanticipated finding. Covert contraceptive use by single women appears to be premised on an anticipation that their relationships is not guaranteed to go long-term. The urge to use contraception secretly and possibly be able to feign pregnancy appears to be a pre-emptive measure taken by single women to minimize their losses in the event of a breakdown in the often male-dominated relationship. This possible explanation is supported by the fact that, at three hundred Ghana Cedis a month, the majority of women, including single women living the municipality do not earn enough to meet the demands of living unsupported in a municipality, and are therefore vulnerable economically [31].

In this study, although women were of the view that using the injectable, IUD and implants better facilitated covert use than the pills, we did not find a significant association between the type of methods women used and whether they used them covertly or otherwise. This finding is in contrast with that of a study in urban Ndola in Zambia where users of the injectable were 4 times likely to be in covert use than users of the OCP (5.4 versus 19.7 %, *P*-value = 0.0004) [1]. Similarly, but rather less definitely, studies in Ghana and some other countries in SSA have attributed the popularity of the injectable to the ease with which it can be used covertly [19, 33, 41]. In another study in Zambia however, women who made contraceptive decisions without involving their spouses were rather 30 % less likely to be using the injectable, long-acting and permanent methods. This somewhat contrasting picture in the pattern of partner involvement and the type of methods women used may be attributed to the fact that in all of these studies there has been a failure to account for the effect of availability and access to the different types of FP methods in the clinics where women seek services. Evidence of how health system and provider factors affect the adoption of particular types of FP methods has been demonstrated in studies across SSA [21, 42, 43]. In future, studies that explore the possible association between the type of FP method women adopt and CCU should give consideration to health system and provider factors that affect availability and access to the various methods.

This findings of this study have highlighted the well-reported disconnect in attitude towards contraceptive use between partners [15, 44]. It is an irony that in spite of the apparently low level of reported spousal communication about contraceptives use, many women report partner disapproval as the reason they practice CCU. The extent of this disproportion suggest that many of such women do not actually discuss the issue with their partners but rather make presumptions of spousal disapproval. As other studies have demonstrated, this presumption may not be always correct as attitudes towards contraceptives use have not uncommonly been found to be similar among couples interviewed separately and then together [18, 45]. Even where differences have been found, couples were less likely to be using a method when the wife wanted to have more children, and more likely to be using one when she wants to stop childbearing [46]. It is well possible therefore that in some situations and contrary to widely-held constructs, a women’s use of a method is dictated by an intrinsic desire and spousal disapproval may be more of perception than reality.

In this urban sample, we found that mass media was the dominant source of information on FP. We consider this to offer leverage for the FP program in Ghana as it creates the possibility for sending messages that are targeted at overcoming specific challenges to FP access and unfretted use. Promotional messages that are designed on the basis of evidence from research into the practice of CCU need to be developed. They should be targeted at increasing knowledge and countering the many myths and misinformation that persist in Ghana about FP [47]. Interventions that increase male involvement need to be mainstreamed in the delivery of reproductive health services to encourage spousal communication and shared decision-making [14, 48]. This is likely to save many women the risk, anxiety and psychological distress associated with CCU. In this study we found that none of the women who indicated that their husbands were the main source of FP information practised CCU (Table 1).

Our study has a number of important limitations. The sample size was not adequate enough to enable in-depth exploratory analysis of the factors that were found to be independently associated with CCU. The inadequacy in the size of the sample also manifested in the wide confidence intervals around some of the estimates in the multivariate model. It also affected analysis of the data on the effect of the main sources of FP information. Interpretation of views expressed in the FGDs should also take into account the fact that some participants were not on any modern FP method at the time of the study. Another limitation was the lack of data on provider and health system factors that may have affected the type of FP methods women ultimately used. The analysis in this regards would have enhanced by the availability of data on the quality of FP services and the availability of the different types of FP methods. An inadvertent error in pre-categorising the ages of respondents at the time of questionnaire design also limited the amount of information that could be generated in that analysis.

## Conclusions

Covert contraceptive use is a sign that providers must continue to take into account women’s rights to confidentiality in FP services [1]. The practice is high in this population and it has disturbing emotional and psychological effects on women. It hinders their ability to optimally exercise their reproductive rights. The health system needs to acknowledge this and institute measures to assist women who wish to practise it to do so safely and assist those who wish to disclose their use to their partners to do so through the offer of couple counselling. The peculiar needs of single women needs to be taken into consideration.

## Abbreviations

CCU, covert contraceptive use; FGDs, focus group discussions; IDIs, in-depth interviews; OCP, oral contraceptive pills; SSA, Sub-Saharan Africa; TFR, total fertility rate
